# Rheology, Non-Isothermal Crystallization Behavior, Mechanical and Thermal Properties of PMMA-Modified Carbon Fiber-Reinforced Poly(Ethylene Terephthalate) Composites

**DOI:** 10.3390/polym10060594

**Published:** 2018-05-29

**Authors:** Guoliang Lin, Dongwei Li, Minyi Liu, Xiaoyi Zhang, Yuying Zheng

**Affiliations:** 1 Fujian Provincial Key Laboratory of Advanced Technology and Informatization in Civil Engineering, Fujian University of Technology, Fuzhou 350118, China; fjptlgl@hotmail.com(G.L.); dwli2005@163.com (D.L.); z7292136@sina.com (X.Z.); 2 College of Chemistry and Chemical Engineering, Fuzhou University, Fuzhou 350116, China; 3 College of Ecological Environment and Urban Construction, Fujian University of Technology, Fuzhou 350118, China; mili302@163.com

**Keywords:** poly(ethylene terephthalate), carbon fiber, PMMA-g-CF, non-isothermal crystallization, rheology

## Abstract

Poly(ethylene terephthalate) (PET) composites containing carbon fiber (CF) or polymethyl methacrylate (PMMA)-grafted carbon fiber (PMMA-g-CF) were prepared by melt compounding. The rheology, non-isothermal crystallization behavior, and mechanical and thermal properties of pure PET, PET/CF and PET/PMMA-g-CF composites were investigated. The results show that the addition of CF or PMMA-g-CF significantly increases the storage modulus (G′), loss modulus (G″), and complex viscosity (η*) of the composites at low frequency. The Cole-Cole plots confirm that the surface modification of CF leads to a better interaction between the CF and PET, and then decreases the heterogeneity of the polymeric systems, which is confirmed by the SEM observation on the tensile fracture surface of the composites. Non-isothermal crystallization analysis shows that the CF or PMMA-g-CF could serve as nucleation agent to accelerate the crystallization rate of the composites, and the effect of PMMA-g-CF is stronger than that of CF. The result is further confirmed by the analysis of the crystallization activation energy for all composites calculated by the Flynn-Wall-Ozawa method. Moreover, the tensile and impact strength and the thermal stability of the composites are improved by CF, while the incorporation of PMMA-g-CF further enhances the tensile and impact strength and thermal stability.

## 1. Introduction

Poly(ethylene terephthalate) (PET), a typical semi-crystalline thermoplastic polyester, has been widely used in industrial films, fibers, and engineering plastics due to its superior physical and mechanical properties. However, it has many drawbacks, such as its slow crystallization rate and high sensitivity to notch impact, and the mechanical properties used for high-performance applications also need to be improved. Thus, many works have been conducted to improve the properties of PET [1,2,3,4,5].

Carbon fiber-reinforced polymer (CFRP) is a kind of advanced composite material that combines the outstanding properties of carbon fiber (CF) with the matrix. Studies on CF-reinforced polymers, such as polypropylene (PP) [6,7], polyamide 6 (PA6) [8,9], poly(butylene terephthalate) PBT [10,11], poly(trimethylene terephthalate) (PTT) [12], poly(ether ether ketone) (PEEK) [13,14], have been widely reported. It is known that the performance of the CF-reinforced polymer is not only dependent on the properties of the reinforced fiber and the matrix, but also on the interfacial adhesion, which determines the way loads transfer from the matrix to the CF. However, the interaction between most of the polymers and carbon fiber without surface treatment is poor due to the non-polar surface of carbon fiber [15]. As a result, many approaches, including electrochemical oxidation [16], chemical grating [17,18], plasma treatment [19], and various other treatments [20,21], have been employed to modify the CFs in order to activate the CF surface or change the microstructure of the CF surface, and then increase the fiber-matrix interfacial adhesion strength. Although PET is also used a polymer matrix in CFRP, few studies have been found regarding the reinforcing mechanism between the modified CF and PET matrix systematically. In addition, there is still a lack of comparative investigation of the effect of CF and surface modification of CF on the structures and properties of the PET matrix.

In this work, the surface of the carbon fiber was modified by chemical grafting of methyl methacrylate (MMA). Then, the CF-reinforced PET composites filled with grafted and ungrafted CF were prepared by melt blending, respectively. The focus of this work was to investigate the effect of CF and the chemical modification of CF on the rheology and non-isothermal crystallization behavior of the composites, and the effective energy barrier of the non-isothermal crystallization process for the composites was also estimated based on the Flynn-Wall-Ozawa method. In addition, the reinforcing and toughening effects of grafted and ungrafted CF on the PET matrix were studied by the means of the tensile and impact testing. Furthermore, the thermal stability of the three samples was also studied by thermogravimetric analysis (TGA).

## 2. Experimental Section

### 2.1. Materials

Poly(ethylene terephthalate), BG-80, was supplied by Sinopec Yizheng Chemical Fiber Co., Ltd. (Yizheng, China). Carbon fiber (CF), with was supplied by Nanjing Weida Composite Material Co., Ltd. (Nanjing, China). The characteristics of CF are given as follows: the average diameter is 15 μm, the average length is 2 mm, the tensile strength is 3.8 GPa, and the tensile modulus is 230 GPa. The benzoyl peroxide (BPO), acetone, nitric acid, and sulfuric acid were purchased from Sinopharm Medicine Holding Co., Ltd. (Shanghai, China). The methyl methacrylate (MMA) was purchased from Xilong Chemical Co., Ltd. (Shantou, China). The polyvinyl alcohol was purchased from Longhai Xiangda Chemical Co., Ltd. (Longhai, China).

### 2.2. Preparation of the Composites

Firstly, the CFs were immersed in acetone for 24 h, followed by repeated washing with distilled water, and dried in an oven for 3 h at 100 °C. Secondly, the treated CFs were then pre-oxidized in a mixed solution of nitric acid and sulfuric acid (1:3, volume ratio) at 60 °C for 3 h. The pre-oxidized CFs were washed with distilled water for four times and then dried in an oven at 66 °C for 2 h prior to the grafting reactions. Finally, 2 mL of 1 wt % aqueous polyvinyl alcohol solution was dissolved in 40 mL distilled water with stirring in a three-neck round-bottom flask at 70 °C for about 1 h, followed by adding 10 g pre-oxidized CFs and the mixture of 0.07 g of free radical initiator benzoyl peroxide (BPO) and 10 mL of methyl methacrylate (MMA). The reaction proceed at 78 ± 2 °C with constant stirring for about 1.5 h. After that, pumping filtration was used to purify the reaction mixture using acetone to remove the non-reacted reactants and the by-products, and then the CF grafted with polymethyl methacrylate (PMMA) (PMMA-g-CF) was obtained.

Prior to use, pure PET, CF, and PMMA-g-CF were all dried in a vacuum drying oven at 100 °C for 12 h, respectively. Then the PET/CF and PET/PMMA-g-CF composites containing with 5 wt % CF and 5 wt % PMMA-g-CF were prepared by melt compounding in a RM-200B torque rheometer at 260 °C for 8 min, respectively. The obtained mixtures were all chopped into small pellets. The pellets were dried in a vacuum drying oven at 100 °C for 12 h, and were then injection molded into various specimens for testing using a JN55-E injection molding machine (Zhenxiong Machinery Co., Ltd., Ningbo, China) with a barrel temperature of 225 to 250 °C and cooled in the room-temperature mold.

### 2.3. Characterization of the Composites

The infrared spectra (IR) of CFs before and after grafting of PMMA was performed on a Fourier transform infrared spectrometer (FTIR) (PE-983G, Perkin Elmer, Palm Springs, CA, USA).

Scanning electron microscopy (SEM) (JSM-7500F, JEOL, Tokyo, Japan) was operated at an operating voltage of 5 kV to characterize the surface morphology of carbon fiber, as well as the tensile fracture surface of PET/CF and PET/PMMA-g-CF composites.

The chemical composition and functional groups of CF and chemical modification of CF were characterized by X-ray photoelectron spectroscopy (XPS) (ESCALAB250, Thermo Scientific, Beverly, MA, USA) using 300 W Al Kα radiation. The base pressure was about 3 × 10^−9^ mbar. The binding energy peaks were calibrated with C1s at 284.6 eV as a reference.

The non-isothermal crystallization behavior of pure PET and its composites was measured by using a differential scanning calorimeter (DSC Q20, TA instruments, New Castle, DE, USA). All operations were performed under a nitrogen atmosphere with a sample weight of about 5 mg. All the samples were first heated to 270 °C and held at this temperature for 10 min to erase the thermal history. Finally, the samples were cooled to 80 °C at different constant rates of 5, 10, 20, and 40 °C/min, respectively. As for the cooling rate of 10 °C/min, the samples were also reheated to 270 °C at the heating rate of 10 °C/min after cooled to 80 °C to characterize the melting and crystallization behaviors of the composites.

The rheological analysis was performed on a rheometer (AR-2000, TA instruments, New Castle, DE, USA) at 260 °C using a parallel plate geometry with a diameter of 25 mm. The dynamic frequency sweep test was carried out between 0.1 and 100 rad/s. All the frequency sweeping tests were performed under linear viscoelastic conditions.

The tensile properties were measured with a mechanical properties testing machine (CMT4104, Shenzhen SUNS Metering Technology Co., Ltd, Shenzhen, China) according to Chinese Standard GB/T1040-2006. Charpy impact strength was measured with an impact tester (ZBC1400-2, Shenzhen SUNS Metering Technology Co., Ltd, Shenzhen, China) according to Chinese Standard GB/T1043-1993. All the data reported were the mean and standard deviation from ten determinations.

Thermogravimetric analysis (TGA) (NETZSCH STA449C, Selb, Germany) was performed in a nitrogen atmosphere in the range of 10–750 °C under a heating rate of 20 °C/min while a flow of nitrogen was maintained at 50 mL/min.

## 3. Results and Discussion

### 3.1. Characteristics of Carbon Fiber Surface

**Figure 1:**
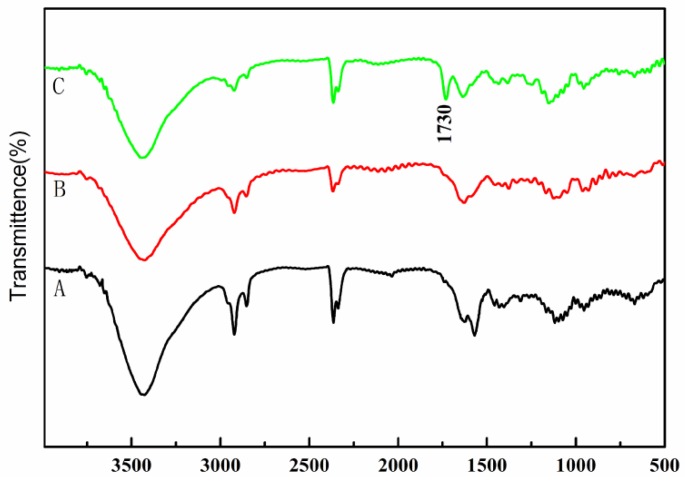
FTIR spectra of (A) untreated CF, (B) nitric acid and sulfuric acid-treated CF, and (C) MMA-treated CF.

The carbon fiber samples before and after treatment were characterized by FTIR. Figure 1 shows the FTIR spectra of treated and untreated CF. As shown in Figure 1, the peaks located at 3430 cm^−1^ originate from the stretching vibration of –OH, and the peak at 2923 cm^−1^, arising from the asymmetric and symmetric stretching vibrations of the C–H bond of the –CH_2_ groups, appeared in the treated and untreated CF. The peak situated at 1730 cm^−1^, appearing in Figure 1C, can be attributed to the characteristic peaks of C=O after the functionalization of MMA. The results suggest that the PMMA has been successfully bound onto the CF surfaces [22].

**Figure 2:**
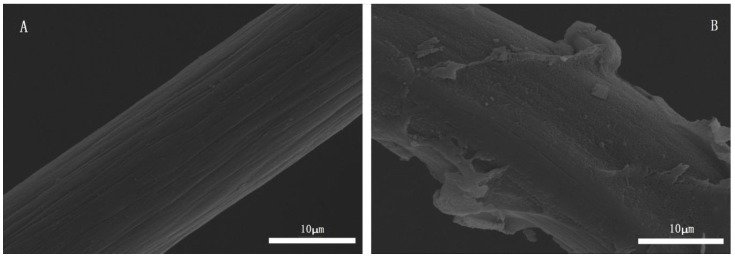
SEM micrographs of (**A**) untreated CF and (**B**) PMMA-g-CF.

The SEM images of untreated CF and PMMA-g-CF are shown in Figure 2. As shown in Figure 2A, it can be observed that the untreated CF with a few narrow grooves is relatively neat and smooth. In contrast, the surface of the PMMA-g-CF in Figure 2B becomes rough and is wrapped by a thick layer of polymer. This indicates that PMMA is chemically grafted on the carbon fiber, generating a layer of PMMA particles on the carbon fiber’s surface. The rougher surface on the grafted carbon fiber is expected to be of benefit to improve the adhesion of grafted CF and the matrix, which then improves the mechanical properties of the composites.

**Figure 3:**
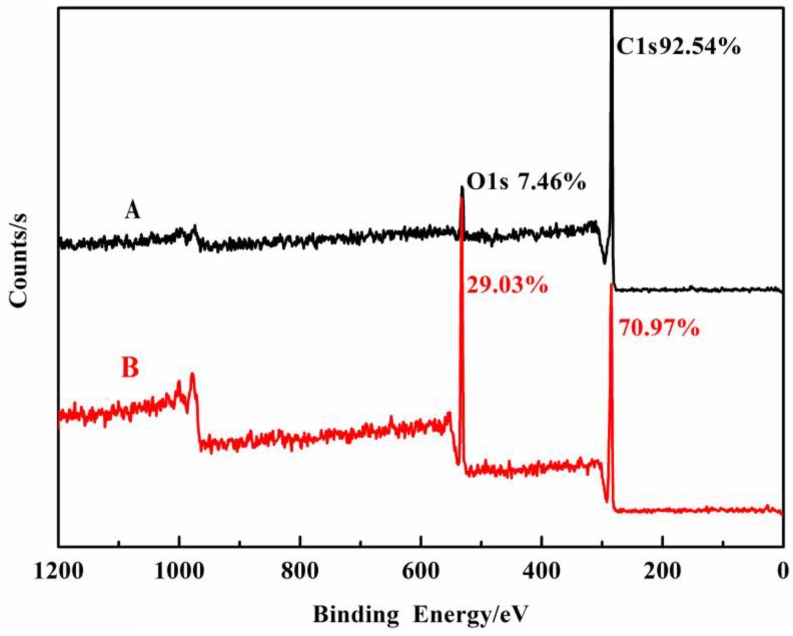
XPS wide scan spectra of (A) untreated CF and (B) PMMA-g-CF.

**Figure 4:**
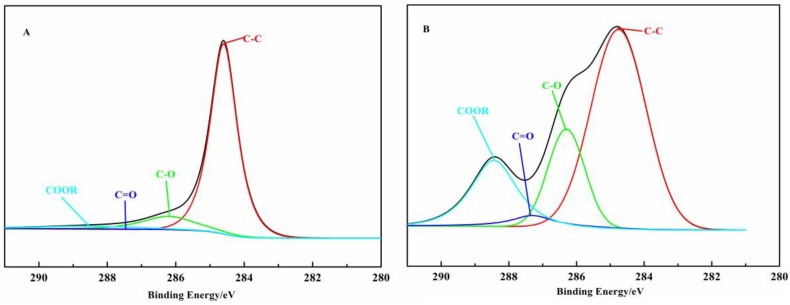
XPS C1s spectra of (**A**) untreated CF and (**B**) PMMA-g-CF.

Figure 3 shows the wide-scan XPS spectra of different elements on the carbon fiber’s surface before and after treatment. As shown in Figure 3, the content of carbon decreases from 92.54% to 70.97% and the content of oxygen increases from 7.46% to 29.03% after the modification of MMA. In addition, the surface atomic O/C ratios increase from 0.08 to 0.41, suggesting the change of the polarity of the CF surface. To further investigate the content of functional groups on the CF surface, the C1s spectra is peak fitted into four peaks and the results are shown in Table 1 and Figure 4. It is clearly seen that the percentages of the C–OH, C=O, and O=C–OR functional groups on the CF surface after modification increases from 11.6%, 1.3%, and 5.2% to 19.3%, 3.5%, and 18%, respectively, suggesting the successful grafting of PMMA.

In short, through the analysis of FTIR, SEM, and XPS results, it can be concluded that the surfaces of CFs have been grafted with PMMA.

### 3.2. Rheological Properties

**Figure 5:**
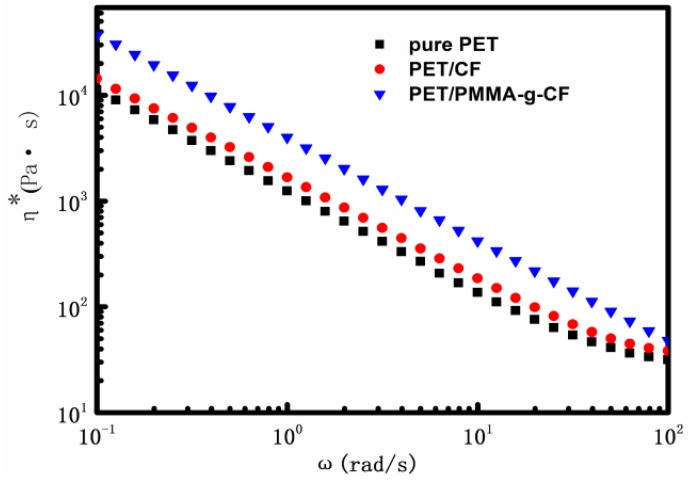
Complex viscosity (η*) vs. angular frequency (ω) for pure PET, PET/CF, and PET/PMMA-g-CF composites.

Figure 5 shows the complex viscosity (η*) of pure PET, PET/CF, and PET/PMMA-g-CF composites vs. the angular frequency (ω). It can be seen that the complex viscosity values of all the samples decrease gradually with increasing ω, exhibiting the pseudoplastic behavior. Meanwhile, the values of η* increase with the addition of CF or PMMA-g-CF within our research scope. This phenomenon indicates that the addition of CF or PMMA-g-CF disturbs the mobility of polymer chains in the melt and then increases the complex viscosity. In comparison with pure PET and the composites, it is clearly seen that the composites with PMMA-g-CF have larger viscosity due to the stronger interaction between the PMMA-g-CF and PET matrix, which is confirmed by the SEM analysis of the tensile fracture surfaces of the PET/PMMA-g-CF composites below.

**Figure 6:**
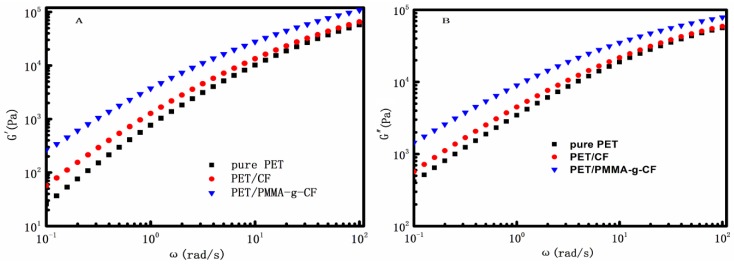
(**A**) Storage modulus (G′), and (**B**) loss modulus (G″) as a function of the frequency of pure PET, PET/CF, and PET/PMMA-g-CF composites.

The storage modulus (G′) and the loss modulus (G″) versus frequency (ω) for all the test samples are shown in Figure 6. It is observed from Figure 6A that the G′ values of the composites increase with the addition of CF or PMMA-g-CF in the whole frequency region, which indicates that the melt strength increases. In addition, the G′ value of the PET/PMMA-g-CF composite is larger than that of the pure PET and PET/CF composite, especially in the low-frequency region. It is well known that the storage modulus represents the elastic response of a material. As shown in Figure 6A, PMMA-g-CF has a significant reinforcing effect on the G′ of the PET, which means that the PET/PMMA-g-CF composite can store large amounts of energy during deformation, resulting in the improvement of the Charpy impact strength. The result is consistent with the mechanical measurements described below. Figure 6B shows the similar trend of G″ to that for G′.

**Figure 7:**
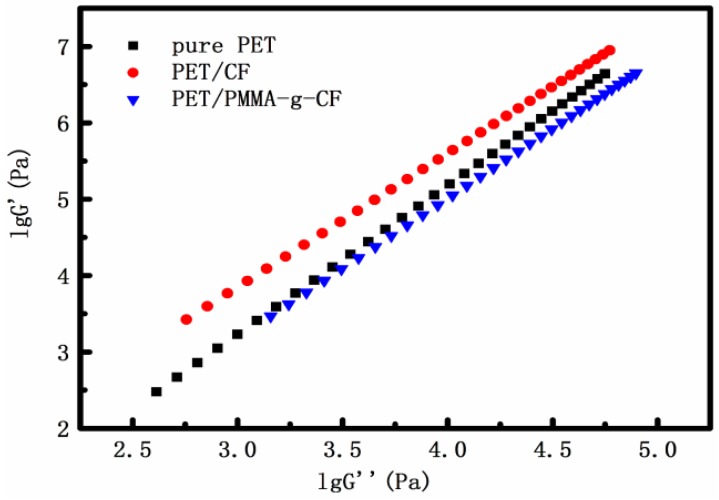
Cole-Cole plot of pure PET, PET/CF, and PET/PMMA-g-CF composites.

It is generally believed that the homogeneity of polymer melts or solutions can be characterized by the plot of G′ versus G″, which is named the modified Cole-Cole plot by Harrell and Nakajima [23], and the slope value of 2 obtained from the G′ versus G″ plot indicates a homogeneous and isotropic polymer solution or melt [24,25,26]. The plots of G′ versus G″ for pure PET, PET/CF, and PET/PMMA-g-CF composites are shown in Figure 7. One can see that the PET/CF and PET/PMMA-g-CF composites exhibit a deviated curve. The introduction of CF decreases the slope producing the deviation from the master curve corresponding to the PET matrix, indicative of the increased heterogeneity in the system. However, the deviated extent of the PET/PMMA-g-CF composite decreases. This phenomenon suggests that surface modification of CF improves the compatibility between the CF and PET, and then decreases the heterogeneity of the polymeric systems.

### 3.3. Thermal Analysis of the Composites

#### 3.3.1. The Non-isothermal Crystallization Behavior and Melting Behavior

**Figure 8:**
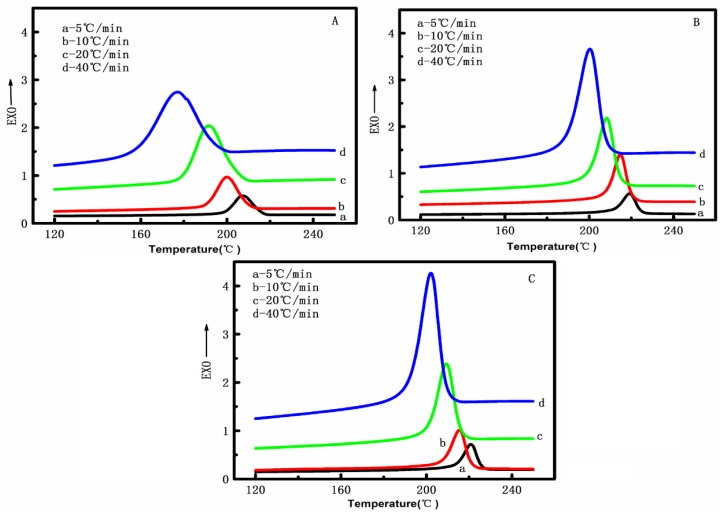
Non-isothermal melt-crystallization exothermal of (**A**) pure PET, (**B**) PET/CF, and (**C**) PET/PMMA-g-CF composites at different cooling rates.

The crystallization curves of pure PET, PET/CF, and PET/PMMA-g-CF composites at various cooling rates (5, 10, 20 and 40 °C/min) are shown in Figure 8. As shown in Figure 8, it is not difficult to find that the curves become wider and the peak temperature (*T*_p_) shifts to the lower temperature as the cooling rate increases gradually. The parameters from the non-isothermal crystallization exotherms for all samples are summarized in Table 2. According to Table 2, it can be seen that the onset temperature of crystallization (*T*_o_) and the peak temperature (*T*_p_) for all samples shift to lower values with the increasing cooling rate. This may be because the crystals have had less time to nucleate and grow as the cooling rate increased. For a given cooling rate, the *T*_o_ and *T*_p_ values of PET are lower than that of PET/CF and PET/PMMA-g-CF composites, which is an indication that the presence of CF or PMMA-g-CF can act as nucleating agent to increase the crystallization rate of PET. This can be attributed to the heterogeneous nucleation effect of the CF or PMMA-g-CF, which facilitates the crystallization of PET chains when the composite is cooled down from the melting temperature.

**Figure 9:**
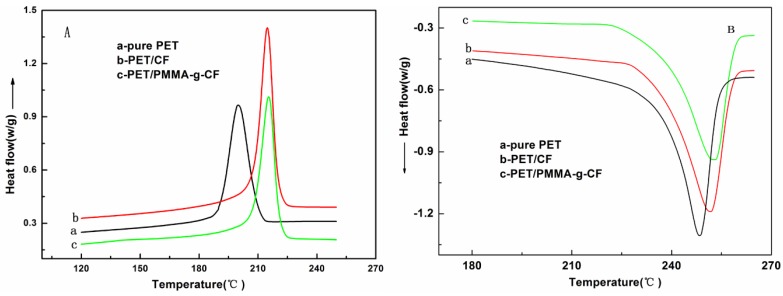
DSC (**A**) cooling and (**B**) the second heating thermograms of PET, PET/CF, and PET/PMMA-g-CF composites.

To better understand the effect of CF and PMMA-g-CF on the crystallization and melting behavior of pure PET, the crystallization and melting behavior of PET/CF and PET/PMMA-g-CF composites are characterized by using DSC cooling and heating thermograms, as shown in Figure 9. The detailed crystallization parameters, such as crystallization temperature (*T*_c_), melting temperature (*T*_m_), and degree of crystallinity (*X*_c_), are shown in Table 3. As shown in Table 3, we can see that the composites show higher *T*_c_ and *X*_c_ values than that of the pure PET, and the values of *T*_c_ and *X*_c_ for PET/PMMA-g-CF composites are higher than that of the PET/CF composites, which can be due to the nucleation effects of the CF and PMMA-g-CF, and then improve the crystallization of the PET matrix. In addition, the PET/PMMA-g-CF composites show higher *T*_m_ values than that of pure PET or PET/CF composites. It can be concluded that higher crystal perfection caused by the PMMA-g-CF results in a higher *T*_m_ of the PET matrix.

#### 3.3.2. Non-Isothermal Crystallization Kinetics 

Generally, the Avrami equation [27] can be used to describe non-isothermal crystallization, and is defined as:

(1) $$1-X_{t}=\exp(-Zt^{n})$$

(2) $$\log[-\ln(1-X_{t})]=\log Z+n\log t$$

where *X*_t_ is the relativity crystallinity at crystallization time *t*, *n* is the Avrami exponent, and *Z* is the crystallization rate constant. Considering the temperature is constantly changing during a non-isothermal process, Jeziorny [28] suggested that the value of the crystallization rate parameter *Z* should be corrected by the cooling rate β as follows:

(3) $$\log Z_{c}=(\log Z)/\beta$$

where *Z*_c_ is the corrected Jeziorny crystallization constant.

**Figure 10:**
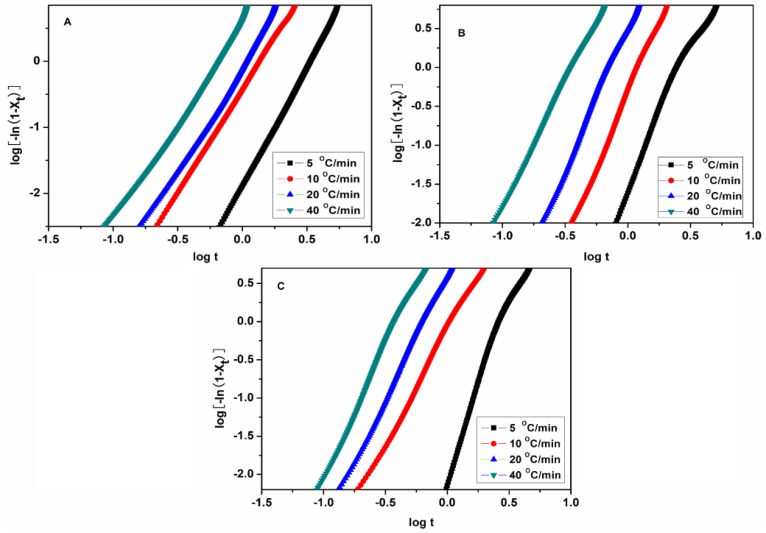
Plots of log[−ln(1 − *X*_t_)] vs. log*t* for non-isothermal crystallization of (**A**) pure PET, (**B**) PET/CF, and (**C**) PET/PMMA-g-CF composites at different cooling rates.

According to Equation (2), the plots of log[−ln(1 − *X*_t_)] against log*t* for PET, PET/CF, and PET/PMMA-g-CF composites are shown in Figure 10. The values of *n* and *Z* can be obtained by the slopes and the intercepts of lines, respectively, and the values of *Z*_c_ can be calculated by Equation (3). The values of *n* and *Z*_c_ are listed in Table 4. As shown in Figure 10, the plots of PET, PET/CF, and PET/PMMA-g-CF composites are fairly linear, although they deviate at the beginning and the end, which is usually attributed to the induced period during initial crystallization and secondary crystallization at the end of crystallization.

It can be seen from Table 4 that the *n* values for pure PET range from 2.76 to 3.32, which means a three-dimensional spherical growth and homogeneous nucleation. After adding the CF or PMMA-g-CF, the *n* values are found to be in the range of 2.72–3.50. This result suggests that the addition of CF or PMMA-g-CF does not significantly change the nucleation mechanism and crystal growth of the PET matrix. In addition, as shown in Table 4, higher *Z*_c_ values are obtained for the PET/CF and PET/PMMA-g-CF composites than those of pure PET under the same cooling rate. Usually, a higher *Z*_c_ value means faster crystallization rate of the matrix at the same cooling rate [29]. Therefore, this behavior shows that the incorporation of the CF or PMMA-g-CF can act as a nucleating agent and increase the crystallization rate of the composites.

#### 3.3.3. Crystallization Activation Energy

Usually, the reliable values of the effective activation energy of the non-isothermal crystallization of polymer can be evaluated by the differential iso-conversional method of Flynn-Wall-Ozawa [30]. The Flynn-Wall-Ozawa equation is expressed as follows:

(4) $${\ln\beta }_{i}=Const.-\frac{1.05E_{a}}{RT_{a,i}}$$

where *R* is the universal gas constant, β_i_ is the cooling rate, *E*_a_ is the activation energy for a certain conversion, and *T*_a,i_ refers to the temperature at a certain conversions and cooling rate. 

**Figure 11:**
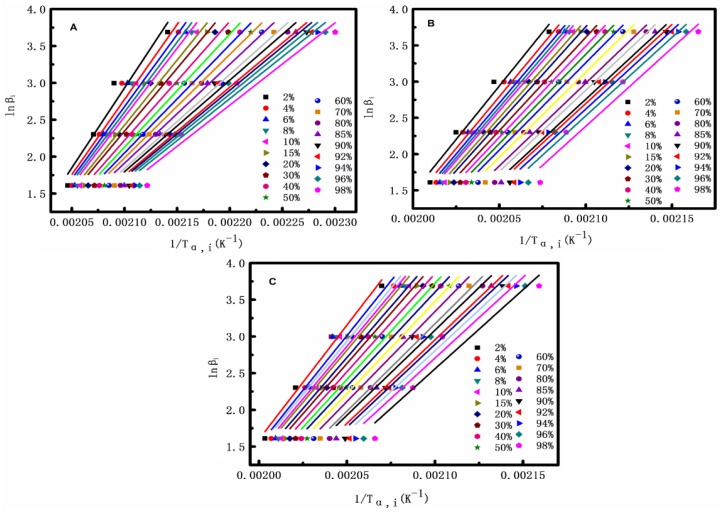
Plots of lnβ_i_ versus 1/*T*_a,i_ for non-isothermal crystallization of (**A**) pure PET, (**B**) PET/CF, and (**C**) PET/PMMA-g-CF composites.

Figure 11 shows the analyses of PET, PET/CF, and PET/PMMA-g-CF composites by the Flynn-Wall-Ozawa method. By plotting of lnβ_i_ against 1/*T*_a,i_, where *i* = 2%, 4%, 6%, 8%, 10%, 15%, …, 90%, 92%, 94%, 96%, 98%, and the *E*_a_ values can be calculated from the slope of the straight lines in Figure 11. 

**Figure 12:**
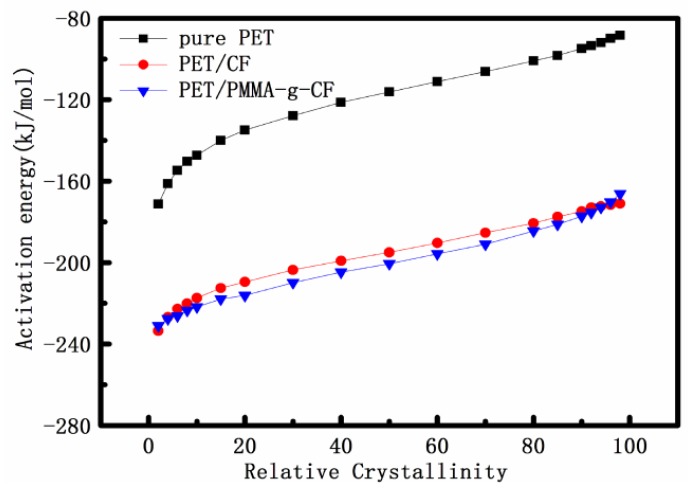
Activation energy as a function of relative crystallinity for pure PET, PET/CF, and PET/PMMA-g-CF composites using the FWO method.

Figure 12 shows the dependence of the effective activation energy *E*_a_ on the relative degree of crystallinity for PET, PET/CF, and PET/PMMA-g-CF composites. It is found that the *E*_a_ values of PET, PET/CF, and PET/PMMA-g-CF composites increase with increasing the extent of the relative crystallization, indicating easier crystallization occurs at lower relative crystallinity. In addition, for a given conversion, the *E*_a_ values of PET/CF and PET/PMMA-g-CF composites are much lower than that of the pure PET, and the *E*_a_ value of PET/PMMA-g-CF composite is lower than that of the PET/CF composite. It is known that the lower the *E*_a_ value, the higher the crystallization ability of the polymer becomes [31]. The results indicate that the addition of CF or PMMA-g-CF has a strong nucleating effect on the PET matrix during the crystallization process, and the effect of PMMA-g-CF is stronger than that of CF due to the enhancement of interfacial interaction between the PET matrix and CF through the surface treatment of PMMA. 

### 3.4. Morphology of the Composites

**Figure 13:**
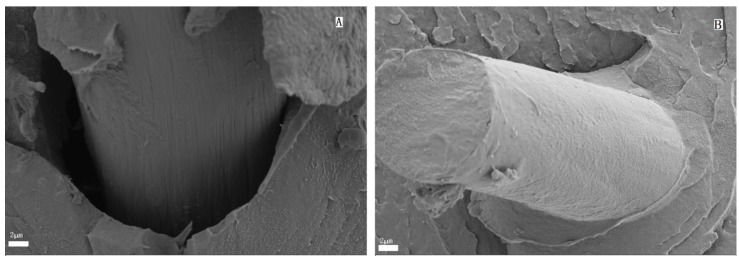
SEM micrographs of the tensile fracture surface of the (**A**) PET/CF and (**B**) PET/PMMA-g-CF composites.

Figure 13 shows the SEM images of the tensile fracture surfaces of the PET/CF and PET/ PMMA-g-CF composites. As can be seen in Figure 13A, the PET/CF composites show an obvious interface region between the CF and the PET matrix, and the surface of the carbon fiber is smooth. These features suggest insufficient adhesion between the untreated CF and the PET matrix. However, it is easy to find that there is a close connection between the PMMA-g-CF and the PET matrix in Figure 13B, which can result in a better interfacial adhesion between the PMMA-g-CF and the PET matrix. The better interfacial adhesion between the treated CF and PET matrix is believed to be of benefit to improve the mechanical properties of the composites.

### 3.5. Mechanical Properties

**Figure 14:**
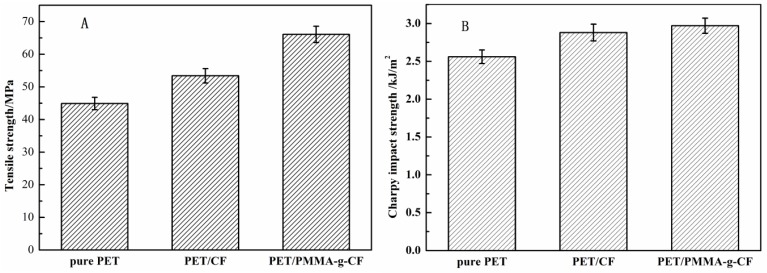
(**A**) Tensile strength and (**B**) Charpy impact strength of pure PET, PET/CF, and PET/PMMA-g-CF composites.

The Charpy impact strength and tensile strength of pure PET, PET/CF, and PET/PMMA-g-CF composites are shown in the Figure 14. As shown in Figure 14, for the CF-reinforced composites, not only does the tensile strength increase with the addition of CF, but the Charpy impact strength also increases. The results imply that the incorporation of CF improves both the rigidity and the toughness as well, which maybe because of the reinforcement effect of the CF. In addition, the addition of PMMA-g-CF further improves the tensile strength and the Charpy impact strength of PET. This may contribute to the addition of PMMA-g-CF increasing the interfacial adhesion between the two phases, which allows for a more efficient stress transfer under stress conditions. The result is consistent with the SEM results above.

### 3.6. Thermogravimetric Analysis (TGA)

**Figure 15:**
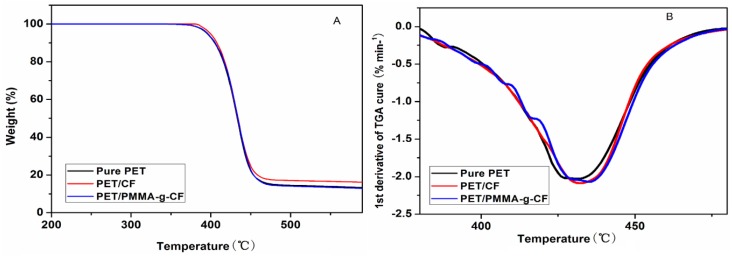
TG (**A**) and DTG (**B**) curves of thermal decomposition of pure PET, PET/CF, and PET/PMMA-g-CF composites.

Thermal stability of the pure PET, PET/CF, and PET/PMMA-g-CF composites is investigated by TGA. The TG and DTG curves of the decomposition temperature for pure PET, PET/CF and PET/PMMA-g-CF composites under N_2_ are shown in Figure 15. As shown in Figure 15, the curves of PET and its composites show one step of mass loss. The decomposition temperature for 10% mass loss (*T*_10%_) and the temperature at the maximum mass loss rate (*T*_max%_) are listed in Table 5. It can be observed in table 5 that the *T*_10__%_ and *T*_max__%_ of the PET are increased with the addition of CF or PMMA-g-CF. The results demonstrate that both the CF and PMMA-g-CF could increase the initial thermal degradation temperature and the thermal stability of PET. In addition, the *T*_max__%_ of PET/PMMA-g-CF composite is higher than that of PET/CF composite, showing higher thermal stability. This behavior could be attributed to the excellent heat stability and thermal conductivity of the surface treatment of CF. The incorporation of PMMA-g-CF into the PET matrix can effectively act as a strong barrier to prevent the diffusion of volatile decomposed products out of the PET/PMMA-g-CF composites during the thermal degradation process.

## 4. Conclusions

In this work, poly(ethylene terephthalate)(PET) composites containing carbon fiber (CF) or polymethyl methacrylate (PMMA)-grafted carbon fiber (PMMA-g-CF) were prepared by melt compounding. The rheology, non-isothermal crystallization behavior, and mechanical and thermal properties of pure PET, PET/CF, and PET/PMMA-g-CF composites were investigated by a parallel-plate rheometer, differential scanning calorimetry (DSC), tensile and impact tests, and thermogravimetric analysis (TGA), respectively. The results show that the addition of CF or PMMA-g-CF significantly increases the storage modulus (G′), loss modulus (G″), and complex viscosity (η*) of the composites at low frequency. The Cole-Cole plots confirm that the surface modification of CF leads to a better interaction between the CF and PET, and then decreases the heterogeneity of the polymeric systems, which is confirmed by the SEM observation on the tensile fracture surface of the composites. Non-isothermal crystallization analysis shows that the CF or PMMA-g-CF could serve as a nucleation agent to accelerate the crystallization rate of the composites, and the effect of PMMA-g-CF is stronger than that of CF. Moreover, based on the analysis of the crystallization activation energy calculated by the Flynn-Wall-Ozawa method, for a given conversion, the values of *E*_a_ for pure PET, PET/CF, and PET/PMMA-g-CF composites decrease in the order of: pure PET > PET/CF > PET/PMMA-g-CF, suggesting that the crystallization rate of PET has been significantly accelerated after the addition of CF or PMMA-g-CF. In addition, it is found that the addition of CF or PMMA-g-CF increases the Charpy impact strength and tensile strength of the PET matrix. TG and DTG curves of pure PET, PET/CF, and PET/PMMA-g-CF composites are shifted to higher temperature. The result indicates the composites have better thermal stability.

## Figures and Tables

**Table 1:** Contents of correlative functional groups on untreated CF and PMMA-g-CF surfaces.

Samples	Contents of Correlative Functional Groups							
	C–C		C–OH		C=O		O=C–OR	
	BE (eV)	%	BE (eV)	%	BE (eV)	%	BE (eV)	%
Untreated CF	284.6	81.9	286.2	11.6	287.4	1.3	288.5	5.2
PMMA-g-CF	284.7	59.2	286.3	19.3	287.3	3.5	288.6	18.0

**Table 2:** Non-isothermal crystallization parameters of pure PET, PET/CF, and PET/PMMA-g-CF composites.

Sample	β (°C/min)	*T*_onset_ (°C)	*T*_p_ (°C)
PET	5	216.29	206.87
	10	209.71	199.87
	20	204.60	191.6
	40	194.11	177.02
PET/CF	5	224.48	218.87
	10	220.11	214.76
	20	214.42	208.31
	40	207.84	200.33
PET/PMMA-g-CF	5	225.78	220.62
	10	220.90	215.45
	20	215.85	209.38
	40	209.01	202.06

**Table 3:** Summary of thermal properties of PET, PET/CF, and PET/PMMA-g-CF composites.

Sample	*T*_c_ (°C)	*T*_m_ (°C)	*X*_c_ (%)
PET	199.87	247.15	33.31
PET/CF	214.76	248.24	35.72
PET/PMMA-g-CF	215.45	248.49	36.69

**Table 4:** Non-isothermal crystallization kinetic parameters based on the Jeziorny-modified Avrami equation.

Sample	β (°C/min)	*n*	*Z*_c_
PET	5	3.32	0.44
	10	2.99	0.83
	20	3.00	0.97
	40	2.76	1.26
PET/CF	5	3.30	0.51
	10	3.34	0.87
	20	3.13	1.22
	40	2.88	1.78
PET/PMMA-g-CF	5	3.50	0.47
	10	2.72	0.95
	20	2.93	1.29
	40	2.99	1.73

**Table 5:** TG and DTG data of pure PET, PET/CF, and PET/PMMA-g-CF composites.

Samples	*T*_10%_ (°C)	*T*_max%_ (°C)
pure PET	405	431
PET/CF	408	433
PET/PMMA-g-CF	406	435
